# Tumor-independent Detection of Inherited Mismatch Repair Deficiency for the Diagnosis of Lynch Syndrome with High Specificity and Sensitivity

**DOI:** 10.1158/2767-9764.CRC-22-0384

**Published:** 2023-03-02

**Authors:** Minttu Kansikas, Laura Vähätalo, Jukka Kantelinen, Mariann Kasela, Jaana Putula, Anni Døhlen, Pauliina Paloviita, Emmi Kärkkäinen, Niklas Lahti, Philippe Arnez, Sami Kilpinen, Beatriz Alcala-Repo, Kirsi Pylvänäinen, Minna Pöyhönen, Päivi Peltomäki, Heikki J. Järvinen, Toni T. Seppälä, Laura Renkonen-Sinisalo, Anna Lepistö, Jukka-Pekka Mecklin, Minna Nyström

**Affiliations:** 1 LS CancerDiag Ltd., Helsinki, Finland.; 2 Faculty of Biological and Environmental Sciences, University of Helsinki, Helsinki, Finland.; 3 Department of Surgery, Helsinki University Hospital, Helsinki, Finland.; 4 Department of Education and Science, Nova Hospital, Central Finland Health Care District, Jyväskylä, Finland.; 5 Department of Genetics, HUSLAB, Helsinki University Hospital Diagnostic Center, Helsinki, Finland.; 6 Department of Medical and Clinical Genetics, University of Helsinki, Helsinki, Finland.; 7 Applied Tumor Genomics, Research Programs Unit, University of Helsinki, Helsinki, Finland.; 8 Faculty of Medicine and Medical Technology, University of Tampere, Tampere, Finland.; 9 Department of Gastroenterology and Alimentary Tract Surgery, Tampere University Hospital, Tampere, Finland.; 10 Faculty of Sports and Health Sciences, University of Jyväskylä, Jyväskylä, Finland.

## Abstract

Lynch syndrome (LS) is the most common hereditary cancer syndrome. Early diagnosis improves prognosis and reduces health care costs, through existing cancer surveillance methods. The problem is finding and diagnosing the cancer predisposing genetic condition. The current workup involves a complex array of tests that combines family cancer history and clinical phenotypes with tumor characteristics and sequencing data, followed by a challenging task to interpret the found variant(s). On the basis of the knowledge that an inherited mismatch repair (MMR) deficiency is a hallmark of LS, we have developed and validated a functional MMR test, DiagMMR, that detects inherited MMR deficiency directly from healthy tissue without need of tumor and variant information. The validation included 119 skin biopsies collected from clinically pathogenic MMR variant carriers (*MSH2*, *MSH6*) and controls, and was followed by a small clinical pilot study. The repair reaction was performed on proteins extracted from primary fibroblasts and the interpretation was based on the MMR capability of the sample in relation to cutoff, which distinguishes MMR proficient (non-LS) from MMR deficient (LS) function. The results were compared with the reference standard (germline NGS). The test was shown to have exceptional specificity (100%) with high sensitivity (89%) and accuracy (97%). The ability to efficiently distinguish LS carriers from controls was further shown with a high area under the receiving operating characteristic (AUROC) value (0.97). This test offers an excellent tool for detecting inherited MMR deficiency linked to *MSH2* or *MSH6* and can be used alone or with conventional tests to recognize genetically predisposed individuals.

**Significance:**

Clinical validation of DiagMMR shows high accuracy in distinguishing individuals with hereditary MSH2 or MSH6 MMR deficiency (i.e., LS). The method presented overcomes challenges faced by the complexity of current methods and can be used alone or with conventional tests to improve the ability to recognize genetically predisposed individuals.

## Introduction

Lynch syndrome (LS, previously hereditary nonpolyposis colorectal cancer; MIM# 120435), is the most common hereditary cancer syndrome in the world, affecting 1 in 100 to 300 individuals (1, 2). Characterized by an inherited mismatch repair (MMR) defect, LS predisposes to a significantly increased, persistent lifelong risk of early onset cancer. Early and accurate identification of LS is crucial, because the diagnosis is associated with decreased morbidity and improved clinical outcome through effective preventive clinical surveillance methods (3, 4). Despite relatively effective methods for conducting clinical follow-up for LS carriers, the challenges in detecting and diagnosing LS persist. High prevalence estimates based on MMR gene variant frequencies in large population datasets have not helped the fact that the majority of LS cases still remain undetected (5–7). The current diagnostic complexity lies within mandatory tumor testing coupled with the need to identify and verify the pathogenicity of a germline sequence variant (8). DiagMMR, the novel test described in the article, detects inherited MMR deficiency from healthy tissue and helps to make a diagnosis with or without tumor and variant information.

Dominantly inherited LS is commonly diagnosed through the occurrence(s) of colorectal and/or endometrial cancer although, the risk for other cancers is also higher than what is seen in the general population (9). Pathogenic variants in the MMR genes, *MSH2* (MIM# 609309), *MLH1* (MIM# 120436), *MSH6* (MIM# 600678), and *PMS2* (MIM# 600259) cause gene-associated cancer risks in their carriers. The average cumulative risk for developing any cancer by age 75 is highest for *MSH2* carriers (81%) and *MLH1* carriers (77%) (4). The *MSH2-* and *MLH1*-associated lifetime risk for developing colorectal cancer, and *MSH2-* and *MSH6*-associated risk for developing endometrial cancer has been estimated to be over 10-fold, compared with the general population (4, 6). Of other LS spectrum cancers, bladder, ureter, kidney, and prostate cancers are more commonly associated with *MSH2* defects, while cancers of the stomach, small bowel, bile duct, pancreas, and ovaries are more frequently associated with *MSH2* and *MLH1* defects (4). However, the cancer penetrance driven diagnosis of LS has inevitably contributed to its underdiagnosis. This is emphasized by the estimation that the majority of pathogenic MMR variant carriers have not yet had cancer, or there has not been an indication of LS based on tumor testing (6, 10). There is also a notable number of suspected LS families, in which a variant has not been found (6, 11).

LS diagnostics utilize protein expression analyses by IHC to indicate MMR deficiency in tumor tissue through the absence of specific MMR protein(s) expression. However, it is important to note that MMR deficiency does not necessarily degrade the protein (12, 13). The other tumor phenotype indicating MMR deficiency is microsatellite instability (MSI), which is also increasingly important for treatment decisions. MSI is, however, not characteristic only of LS but of approximately 15%–20% of sporadic colorectal cancers and up to 45% of sporadic endometrial cancers as well (14–17). *MLH1* promotor hypermethylation, which is typically causing MLH1 expression loss and high MSI (MSI-H) in sporadic tumors, is not commonly observed in LS tumors (10). Thus, a hypermethylation analysis can be used to distinguish between hereditary and nonhereditary *MLH1* loss in a tumor. Germline sequencing is used to confirm the presence or absence of a hereditary MMR gene variant. However, it does not always provide a definite LS diagnosis because a single sequence variant may not be found or the pathogenicity and clinical relevance of the found variant(s) are uncertain (18, 19). To help the interpretation of sequencing results, reported variants are classified in line with the International Agency for Cancer Research classification system and The American College of Medical Genetics and Genomics (ACMG) and Association for Molecular Pathology recommendations, as pathogenic (class 5), likely pathogenic (class 4), variants of uncertain significance (VUS; class 3), likely benign (class 2), or benign (class 1). However, currently 65% of *MSH2* (1,243/1,932), 86% of *MSH6* (1,410/1,649), 94% of *MLH1* (1,684/1,788), and 85% of *PMS2* (799/938) unique variants listed in the International Society for Gastrointestinal Hereditary Tumours (InSiGHT) database are submitted as VUS or have no classification assigned to them (20). So far, 1,714 nontruncating variants have undergone the InSiGHT expert panel review of multiple points of variant effect information, including available clinical and functional data, but the task is challenging and extends to variants uploaded to the U.S. NCBI's ClinVar database (19, 21).

Overall, studies have shown that with the current methods, the adherence to LS screening recommendations is limited (5, 22). As such, large-scale genetic screenings have not been predicted to be effective (23), while molecular testing of colorectal cancer and endometrial cancer tumor tissue has been shown to be cost-effective, particularly for individuals <70 years old (23, 24). Still, LS diagnostics lack a predictive stand-alone method, which could change the course toward proactive recognition and surveillance of LS carriers prior to cancer appearance; a method for accurate LS identification, while also relieving non-LS families and non-carriers in LS families from lifelong clinical follow-up programs.

Here, we introduce a novel LS carrier test called DiagMMR and demonstrate its exceptional high specificity and sensitivity to detect individuals who have an inherited MMR deficiency linked to the MSH2 or MSH6 proteins*.* The functional MMR defect is detected from skin fibroblasts, enabling its use for preventive and early LS identification. DiagMMR offers a novel single test tool to improve the current LS diagnostic workup, cancer prediction, and prevention strategies.

## Materials and Methods

### Study Design and Sample Collection

The purpose of the study was to clinically validate the DiagMMR test and determine its specificity and sensitivity for *MSH2* and *MSH6* caused LS detection. Study included people who had been confidently identified as LS cases. Thereafter, we demonstrated the utility of DiagMMR in a small clinical pilot study including individuals whose LS diagnosis had remained inconclusive. Because of the lack of other diagnostic functional methods, we used sequencing as a reference test for the method validation. While sequencing does not reveal function of proteins, it is considered the gold standard test for LS diagnosis. DiagMMR test results were interpreted either as normal (proficient) MMR or reduced (deficient) MMR based on the MMR capability of the sample in relation to cutoff, which distinguishes MMR proficient from MMR-deficient function.

Sample collection was organized at the Helsinki University Central Hospital (HUCH) and Jyväskylä Central Hospital, Finland. For the clinical validation, skin samples were collected from LS pathogenic variant carriers during their colonoscopy control visits, from controls, and from LS family members who had been called for genetic counseling as risk members but did not know their own carrier status yet. From them, the skin samples were collected after genetic counseling visit and tested in parallel to blood sample collection and sequencing (performed at HUCH). A separate postvalidation pilot cohort included seven LS suspected individuals based on tumor studies but whose sequencing result was either inconsistent or the variant was not found.

Samples were collected with written informed consent from all participating individuals and ethical approval from local Institutional Review Boards and relevant operative units of participating hospitals [Dnro 466/E6/2001 (HUCH) and amendments 17.12.2008 and 12.12.2012, Dnro 28/2003, 7.2.2016]. The study was conducted according to the guidelines of the Helsinki Declaration.

### Clinical Validation Samples

**FIGURE 1:**
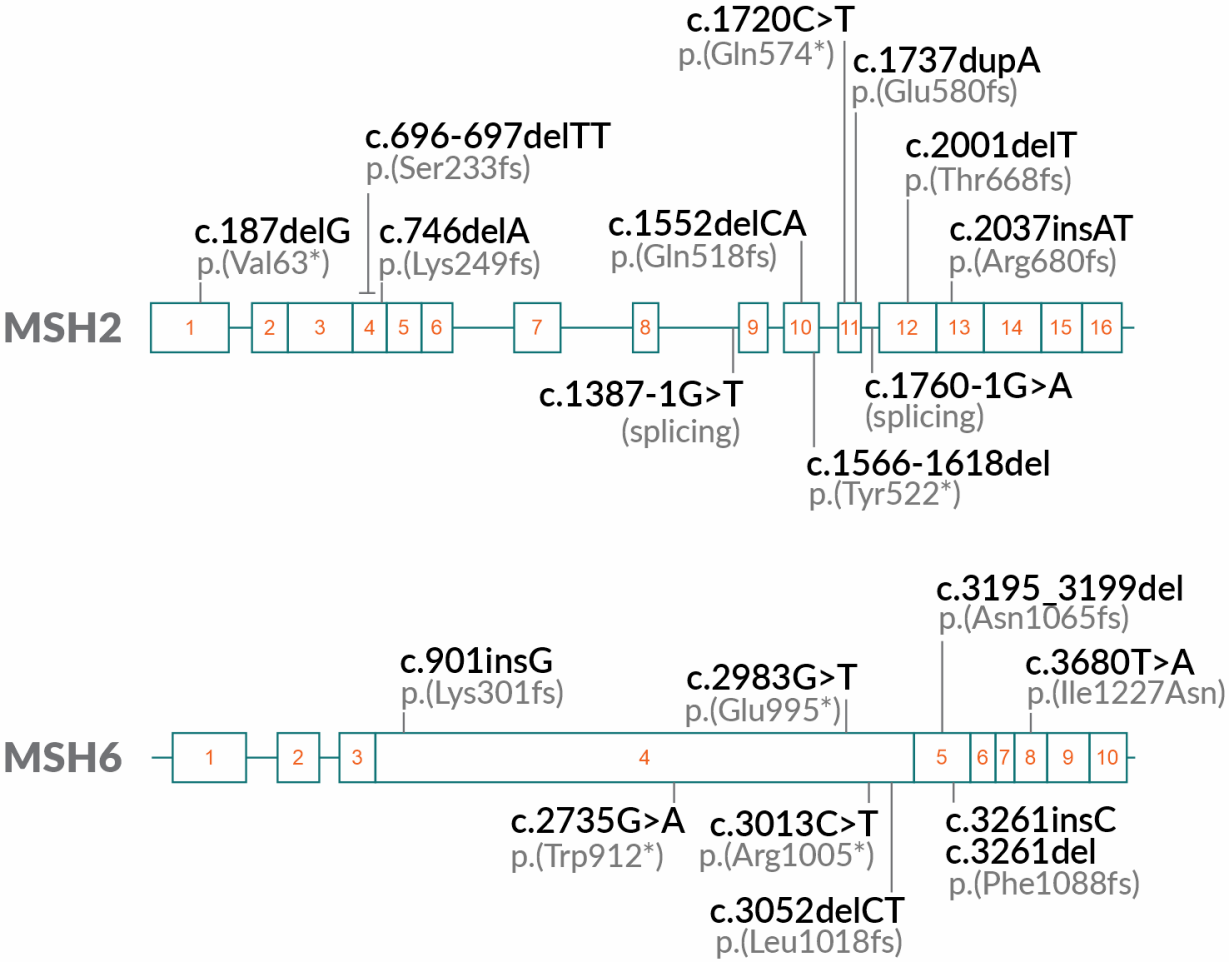
The 20 unique variants found from LS carriers included in clinical validation.

Altogether, 119 skin samples were collected for the validation study. This cohort included samples from carriers (*n* = 28) whose variant in *MSH2* or *MSH6* was confirmed by sequencing (LS carrier), and controls not suspected to carry a germline pathogenic variant (*n* = 65) or in whom a pathogenic variant segregating in their LS family was not detected by germline sequencing (*n* = 26). Of the 28 carrier samples, 17 were from *MSH2* and 11 from *MSH6* variant carriers. Among the carriers, 20 different pathogenic variants (11 in *MSH2* and 9 in *MSH6*) were dispersed along the genes and represented a range of typical LS-associated alterations affecting different functional domains along the proteins (Fig. 1). The InSiGHT LOVD database was further used (see Discussion) for determining the pathogenicity of variants included in the study (20).

### DiagMMR

**FIGURE 2:**
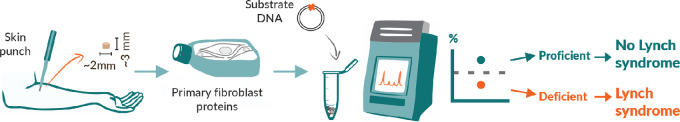
The DiagMMR method for determining MMR efficiency from constitutional cells.

All samples were routinely treated according to the DiagMMR protocol outlined in Fig. 2. The DiagMMR method has been previously optimized with 259 skin samples for the detection of MMR deficiency caused by an inherited pathogenic variant in the *MSH2* or *MSH6* gene. The method includes the entire process from sampling to fibroblast culture, functional MMR efficiency testing, and results interpretation.

### Sampling and Fibroblast Culture

All dermal biopsies were collected from inner forearm using a ≥2 mm biopsy at 2–3 mm depth. Samples kept at room temperature were dissected within 4 days for primary cell culture. To promote tissue adherence and cell growth, tissue dissections were dried to a cell culture flask in humid conditions. After 14 days of initial cell growth in standard fibroblast cell culture conditions, cells were trypsinized off the vessel and expanded in subsequent culture to approximately 1 × 10^8^ cells.

### Protein Extraction

Nuclear proteins were extracted as described previously (25). In brief, cells were collected and treated with cold isotonic and hypotonic buffers prior to the disruption of cell membranes. Nuclei were then centrifuged prior to the desalting of the protein sample extracted. Nuclear proteins were quantified using Qubit 3.0 fluorometer (Thermo Fisher Scientific, RRID:SCR_020311).

### MMR Efficiency

To distinguish between MMR proficiency and deficiency, the functional MMR efficiency was assessed as described previously (25, 26). Briefly, 80 μg of nuclear proteins were incubated at 37°C with 50 ng of circular GT heteroduplex substrate for 1 hour. The reaction was terminated with proteinase K and clarified of proteins with phenol:chloroform:isoamyl alcohol prior to substrate DNA precipitation and subsequent restriction analysis with *Eco*31I and *Bgl*II. The *Bgl*II enzyme is specific for repaired substrate only and allows the quantitation of MMR repair in each reaction. Repair efficiency was visualized with gel electrophoresis using a 1% agarose gel ran at 100 V for 1 hour and quantified by GeneTools (SynGene, RRID:SCR_005663). Repair efficiency is measured as a percentage of repaired DNA of total amount of DNA in each reaction.

### DiagMMR Result Interpretation

A DiagMMR was used to quantify the MMR capability of the sample. Results interpretation relies on a cutoff demonstrated to distinguish MMR proficient (MMR normal, non-LS) from MMR deficient (MMR reduced, LS) samples. To minimize the potential for false-positive (FP) results, the cutoff has been set at the lowest repair efficiencies measured when testing 12 control samples in 37 individual reactions. On the basis of optimizations and the quantification resolution, the cut-off area was determined to be ±5%. Samples, which repeatedly show MMR efficiency near the cut-off level are interpreted as “gray area” samples indicating intermediate level of MMR efficiency, neither normal MMR, nor that reduced MMR level, which was shown to be typical to clearly pathogenic MMR variant carriers. Because the test result is quantitative, it is natural that the repair efficiencies also slightly vary from test to test for the same sample. Thus, the interpretations were done based on at least two independent assay results.

### Statistical Analysis

Statistical analyses were performed with Clopper–Pearson and standard logit confidence intervals using MedCalc Software (RRID:SCR_015044; ref. 27). ROC analysis and the AUC calculation was made for samples interpreted as MMR proficient and MMR deficient with R (RRID:SCR_001905; ref. 28) version 3.6.2 by using library plotROC. Confidence interval calculation was based on Clopper and Pearson (29) and Pepe (30) as per plotROC implementation.

### Postvalidation Clinical Pilot

After the clinical validation study, the DiagMMR method was used to test seven LS suspected individuals for whom conventional diagnostic methods (IHC analysis of tumor tissue followed by panel sequencing and multiplex ligation-dependent probe amplification (MLPA) of blood DNA) had not given a definitive diagnosis. Patients were selected and skin samples obtained at the Helsinki University Central Hospital. DiagMMR testing and result interpretation was done without knowledge of the clinical phenotypes and previous test results of the individuals. Only after the DiagMMR result delivery to clinicians, the results were compared with other clinical information (see Results, Table 2). Patient 1, with two metachronous LS spectrum adenocarcinomas showing MSH2 loss in IHC, cancer diagnosed prior to age 60 and a first-degree relative with four primary malignancies, was found to be a carrier of *MSH2* 1805T>C (p.Leu602Pro). The variant was classified as VUS at the time of genetic diagnosis. Tested individuals 2 and 3 are direct descendants of patient 1, both cancer free at age 30, the latter shown to carry the *MSH2* 1805T>C VUS. Patient 4 has been diagnosed with rectum adenocarcinoma at the age of 71. Despite the lack of family history information and that no variants were found with a 12-gene next-generation sequencing (NGS) panel including *MSH2* and *MSH6*, the tumor tissue was shown to be MSH2 and MSH6 negative by IHC. Patients 5 and 6 with rectum and uterine carcinoma, respectively, diagnosed at age 70–75 with no variants found by NGS, were shown to be MSH6 negative by IHC. The grandparent of Patient 5 has been diagnosed with gastrointestinal cancer and Patient 6's family history demonstrates multiple LS-spectrum cancers in first- and second-degree relatives. Patient 7 has been diagnosed with MSH2-negative colon carcinoma at the age of 33, but no sequence variant has been identified. Numerous tumors had been diagnosed in Patient 7’s first-degree relatives.

### Data Availability

Raw data for this study were generated at the University of Helsinki (Helsinki, Finland) and LS CancerDiag Ltd (functional data) and Helsinki University Hospital (clinical data) and are not publicly available due to patient privacy requirements. Derived data (Tables 1 and 2) supporting the findings of this study are available within the article.

**TABLE 1:** Clinical validation of DiagMMR

	Reference standard, LS carrier	Reference standard, No LS	
DiagMMR positive	24 (True positives)	0 (False positives)	24 (DiagMMR positives)
DiagMMR negative	3 (False negatives)	90 (True negatives)	93 (DiagMMR negatives)
TOTAL	27	90	117

**TABLE 2:** The clinical information and test results of the patients tested in the clinical pilot project

Patient	Tumor/age of onset	IHC	Sequencing	Family	Other	DiagMMR
1	Colon ad.ca./50	MSH2-	*MSH2* 1805T>C (p.Leu602Pro)	Parent with multiple LS spectrum tumors by age 79	MSI-H	MMR Reduced
	Prostate ad.ca./57	MSH2-			MSI-H	
2	None/29		*MSH2* normal		Child of patient 1. Colonoscopy normal	MMR Normal
3	None/28		*MSH2* 1805T>C (p.Leu602Pro)		Child of patient 1. Colonoscopy and gastroscopy normal	MMR Reduced
4	Rectum ad.ca./71	MSH2- MSH6-	NGS normal	None confirmed		MMR Normal
5	Rectum ca./71	MSH6-	NGS normal	Grandparent with gastrointestinal cancer		MMR Normal
6	Uterine ca./69	MSH6-	NGS normal	Sibling with gastrointestinal cancer, parent with bladder cancer, second-degree relative with ovarian cancer		MMR Reduced^a^
7	Colon ca./33	MSH2-	NGS normal	Parent with rectal and kidney cancer, child and sister with early onset breast cancer	MSI-H *MSH2*, *MSH6* and *EPCAM* normal MLPA	within cutoff^b^

Abbreviations: Ca., carcinoma; ad.ca, adenocarcinoma; IHC, tumor immunohistochemistry shown protein absence/deficiency; MSI-H; microsatellite instability high tumor; NGS; next-generation sequencing with >95% exonic coverage of *APC, BMPR1A, MLH1, MLH3, MSH2, MSH6, MUTYH, PMS2, POLD1, POLE, SMAD4* and *STK11*.

^a^Indicative result, result not repeated.

^b^Result not indicating significant MMR deficiency.

## Results

### Clinical Validation and Diagnostic Accuracy of DiagMMR

**FIGURE 3:**
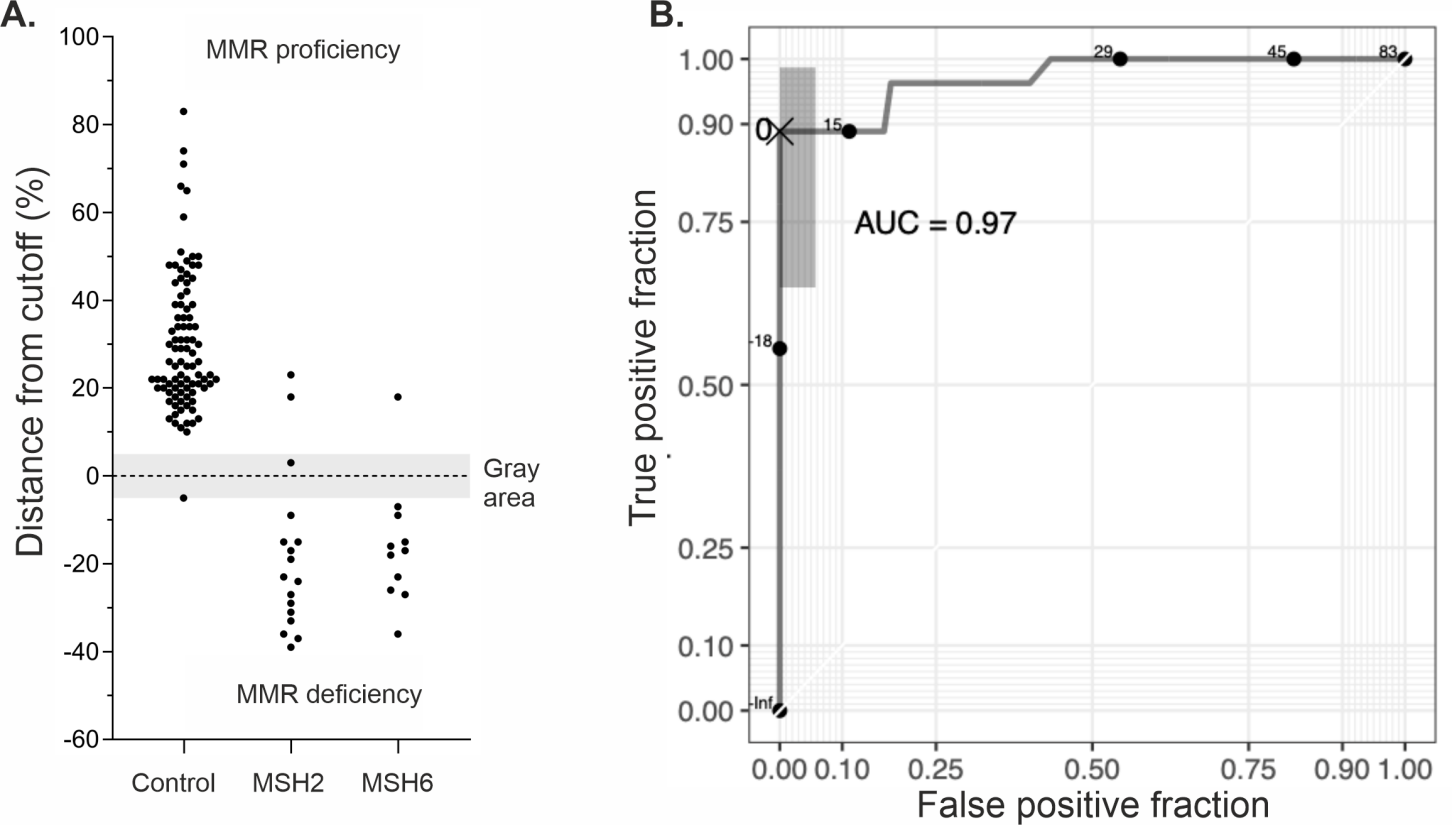
DiagMMR clinical validation results and diagnostic accuracy. **A,** Average positioning of all samples interpreted as MMR proficient (above cutoff), in “gray area” not showing significant proficiency or deficiency (within cut-off area), or MMR deficient (below cutoff; *n* = 119). All results are averages of at least two independent tests yielding a conclusive interpretation. Samples have been separated into three clusters according to sample type on the *x*-axis (controls, MSH2 carrier samples, and MSH6 carrier samples) **B.** ROC curve and corresponding AUC value for prediction of MMR deficiency with DiagMMR. Shaded area corresponds to significance level of 0.01 around cutoff 0 based on Clopper and Pearson (29) and Pepe (30).

In total, 119 dermal biopsies were tested for DiagMMR clinical validation. Of the 119 samples, 93 demonstrated inherited functional MMR proficiency (no-LS) and 24 MMR deficiency (LS) by DiagMMR, while two samples were repeatedly within 5% of the cutoff, inside the “gray area” (Fig. 3A). Comparing the DiagMMR test interpretations obtained for 98% (117/119) of the tested samples with the LS carrier statuses as per the reference standard (Table 1), 90 control samples (90/90) were shown to be MMR proficient, demonstrating no FP and hence yielding an assay specificity of 100% [95% confidence interval (CI), 96.0–100]. Similarly, for carriers of *MSH2* or *MSH6* variants, 24 of 27 samples were shown to be MMR deficient, yielding an assay sensitivity of 88.9% (95% CI, 70.8–97.7). The test accuracy demonstrates that 97.4% (114/117) of the time the test will reveal a result comparable with the reference standard.

The ROC curve for the 117 samples (excluding two gray area samples) confirms the previously defined cut-off level to be optimal (Fig. 3B). The test performance data indicate that increasing sensitivity from current level would rapidly increase FP fraction and therefore the selected cut-off level can be considered optimal. The AUC value reaches an excellent level of 0.97 indicating high overall accuracy.

### Postvalidation Clinical Pilot

Immediately after clinical validation, seven individuals whose LS status had remained inconclusive, were studied with DiagMMR (Helsinki University Central Hospital). After testing the blinded samples, the DiagMMR test results were compared with the available clinical data, which revealed that in cases where tumor tissue had shown MSH2 and/or MSH6 protein expression loss with IHC, an MMR gene variant was found in only one case, and here it is pathogenicity could not be confirmed (Table 2).

**FIGURE 4:**
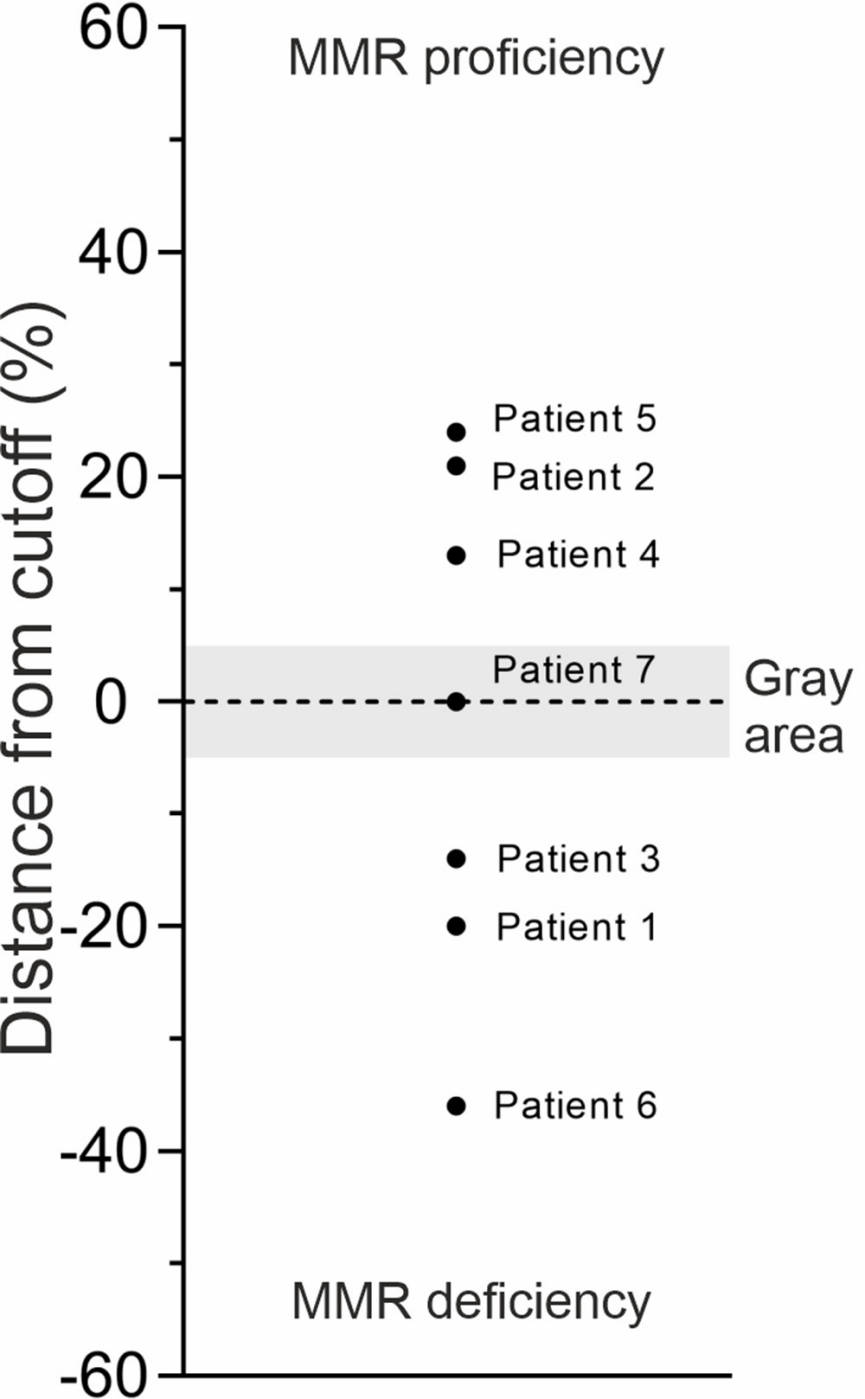
Scatter chart demonstrating the average positioning of the DiagMMR samples included in the clinical pilot. Validated cut-off classifies samples either as MMR proficient (above cutoff, *n* = 3), MMR deficient (below cutoff, *n* = 3) or samples not showing significant proficiency or deficiency (in the gray area, *n* = 1). All results are averages of at least two independent tests yielding a conclusive interpretation, except for patient 6 for whom the result is indicative due to the lack of assay repetitions.

The DiagMMR results of the clinical pilot samples are illustrated in Fig. 4. Patient 1 with *MSH2* 1805T>C (p.Leu602Pro) and two metachronous LS spectrum cancers was shown to have MMR deficiency by DiagMMR. Of the two children, both still healthy, the one whose DiagMMR result showed MMR deficiency had inherited the same variant of uncertain significance (Patient 3) while the child with normal DiagMMR was not a carrier of the VUS (Patient 2). Patients 4 and 5 with rectum cancers at age 71 demonstrated normal MMR efficiency, while Patient 6 with multiple LS spectrum cancers in first- and second-degree family members and the loss of MSH6 expression in her endometrial cancer tissue was shown to have MMR deficiency based on DiagMMR. Patient 7, with early onset colorectal cancer showing MSH2 expression loss but no pathogenic germline variants repeatedly showed MMR efficiency near the cut-off level. The DiagMMR results contributed directly to the personal follow-up plan of the tested individuals by prompting regular colonoscopies for individuals with reduced MMR.

## Discussion

Accurate identification of LS families and at-risk family members, and the exclusion of individuals and families not affected by the condition can have an enormous impact on maintaining health and quality of life. The functional DiagMMR method finds inherited MMR deficiency indicating LS prior to cancer and can hence be placed anywhere in the LS diagnostic pathway. Here, we first demonstrate the accuracy of the test by validating it with 119 samples including LS carriers of pathogenic *MSH2* and *MSH6* variants. The clinical validation was started with *MSH2* and *MSH6*, whose protein decrease was previously shown to strongly affect the MMR efficiency (31). The clinical benefit of the test was further examined through a small pilot study where DiagMMR assisted the diagnosis and clinical management of the tested patients by directing all MMR-deficient patients to regular clinical surveillance.

The global LS prevalence has been reported to be up to 1:100–300, while among patients with endometrial cancer and colorectal cancer even 1:35–40 (2, 6). Consequently, testing of all colorectal cancers and endometrial cancers for MMR deficiency and LS is currently recommended in United States and Europe, but compliance and strategies vary even between hospitals within a single country (32–38). While the occurrence is unanimously estimated to be notable, several studies have tried to estimate the percentage of cases where no MMR gene variant is found with current methods. Indeed, several studies suggest that the percentage of LS not found may be as high as 53%–60% (39–41). Similarly, in up to 30% of tumors showing MSH2 and MSH6 losses in IHC, no germline variant was found, while 30% of all unique MMR gene variants found are reported as VUSs (19, 42). To top it all off, the number of variations left unreported is unknown. The inability to confirm LS through the identification of a pathogenic variant highlights the limitations of diagnostics, but the patient selection methods prior to sequencing play a role too. In fact, a recent study in the United Kingdom demonstrated that nearly half of germline variants would have been missed due to the National Health Service criteria used to select patients for gene panel testing (43). While tumor IHC and MSI analyses are particularly sensitive and recommended for colorectal cancer screenings, their sensitivity and specificity vary greatly across different tissue types and require histopathologic expertise within the clinical setting (44, 45). IHC inability to assess the functionality of the expressed protein contributes to its false-negative rate (nonfunctional protein expressed), while changes restricted to the tumor tissue reflect the FP rate. This is particularly relevant for pathogenic missense variants as well as for double somatic MMR gene defects which are particularly common in endometrial cancer and are at least part reason for the challenges in detecting LS in patients with tumor IHC and MSI findings (12, 46). MSI analyses have proven extremely informative for tumor treatment decisions but are unable to distinguish LS tumors from sporadic colorectal cancers with MSI due to *MLH1* promotor hypermethylation (47). In all cases though, current LS diagnostics rely heavily on tumor-derived information and sequencing, with deficiencies in the detection of large deletions and insertions in the coding region and base errors occurring in deep intronic sequences and noncoding regulatory regions (48). Thus, the need for functional assays in translating sequence alterations into clinically actionable diagnoses persist (49–51). In LS, the mechanism causing a high risk for cancer is the dominantly inherited MMR deficiency, no matter where in the genome the pathogenic variant is, what type it is, and whether the protein is degraded or not in a tumor. To our knowledge, DiagMMR is currently the only validated and CE marked functional test for LS (IVDD 98/79/EC). The method is based on our previous work with the functional *in vitro* MMR assay (26) used for the functional assessment of recombinant MMR proteins. The *in vitro* MMR assay was further developed taking advantage of our research results showing that the functional assessment of MMR gene variants and nuclear proteins of cells with reduced MMR gene mRNA expression is effective in recognizing reduced MMR function (25, 31, 52–53). The novelty and inventiveness of the DiagMMR test lies in the fact that it can measure the DNA mismatch repair capability directly from the proteins extracted from the individual's primary fibroblast cells. The test specifically detects the weakening of MMR function in normal human cells. However, the test does not show that this inherited impairment of MMR efficiency in noncancerous cells, although the susceptibility is known to exist, leads to malignancy or other biological consequences without requiring other events in the cell. Although the use of blood cells is generally considered convenient in diagnostics, their suitability for DiagMMR testing has not been demonstrated.

Using clinical diagnoses confirmed by sequencing as a reference standard for identifying *MSH2* and *MSH6* variant carriers, we show that DiagMMR has exceptionally high specificity (100%, no FP), sensitivity (89%), and accuracy (97%). The high AUC value of 0.97 demonstrates the methods ability to detect LS. The lack of FP results is expected as the assay cutoff was based on the lowest repairs measured in a set of control samples to aim for high specificity. Indeed, all controls were interpreted as MMR proficient (normal), except for one which could not be classified as MMR proficient due to its proximity to the cutoff. This and an *MSH2* carrier sample also positioning within 5% of the cutoff were interpreted as samples neither MMR proficient nor deficient. Overall, individuals included in the clinical validation carried 20 different *MSH2/MSH6* variants, which all were interpreted as pathogenic in the hospital. Twelve of those were classified in the InSiGHT LOVD database as pathogenic [10 pathogenic (class 5) and 2 likely pathogenic (class 4)], two were listed as pathogenic but not confirmed, and six clinically categorized pathogenic variants were not listed in the database (20). Of the 27 *MSH2/MSH6* carrier samples, three demonstrated MMR proficiency above the cutoff indicating normal MMR. One of these three variants (*MSH2* c.2001delT) was not listed in the InSiGHT database and here, only one of the two *MSH2* c.2001delT variant carriers belonging to the same family showed deficient MMR function. Similarly, a sample from one *MSH6* c.3013C>T carrier was MMR proficient, while another from an unrelated carrier was MMR deficient. These discrepancies may represent the shortcoming of the assay sensitivity or suggest that while the variant is found in the family member(s), it might not confer cancer susceptibility at all or might not confer it on its own. Indeed, while clinical LS diagnosis of the families is likely correct, the presence of another causative variant segregating in the family can be difficult to rule out, as we have previously shown with a putative *MSH2* LS family whose MMR deficiency was functionally shown to be caused by another *MSH6* variant segregating in the family (54). The *MSH2* c.1720C>T variant was found to be MMR proficient.

The ability to detect LS carriers of pathogenic *MSH2* and *MSH6* variants without tumor data, presents a novel approach for identifying LS. Functionally, the importance of MSH2 and MSH6 is through the mismatch error recognizing and binding heterodimer, MutSα, and its role in recruiting downstream factors including MMR components MLH1 and PMS2 (forming MutLα heterodimer) that are required for the tetrametric promotion of DNA error excision and DNA resynthesis (55). Sequence alterations in MutSα represent 54% of those described in LS susceptibility genes (20). Among LS gene variant carriers, *MSH2* carriers have highest, up to 84% risk of cancer, while the risk for *MSH6* carriers is up to 62% (56). The up to 70% risk for endometrial cancer is the highest for *MSH6* and *MSH2* variant carriers with an over 60% risk to develop a second primary cancer, of the colorectum most commonly (56–58). While the cancer risk associated with pathogenic *MLH1* variants is significant, studies have shown that the MMR mechanism is not as sensitive to expression decrease in the *MLH1* gene as it is to those in *MSH2* and *MSH6* genes (31) This could imply that the cancer risk associated to *MLH1* is more sensitive to other cellular activities, such as DNA damage signaling shown to be more sensitive to decreases in *MLH1* expression than the MMR mechanism (59). However, now that the functionality of the DiagMMR test to detect LS carriers of pathogenic *MSH2* and *MSH6* variants has been successfully proven, optimization and validation studies have been able to start for *MLH1* as well.

Following the validation, we demonstrated the clinical application of the test to assist the diagnosis of seven LS suspected patients with abnormal MSH2 and/or MSH6 findings in the family. Five of these patients had presented with LS spectrum tumors with MSH2 and/or MSH6 deficiency by IHC. However, the diagnosis had not been confirmed as a pathogenic MMR gene variant had not been found. Fittingly, without knowledge of the clinical information at the time of testing, the DiagMMR results showing deficient MMR correlated well with patients with multiple cancers in the family and MSI-H tumor phenotype, while the samples shown to be MMR proficient were from individuals with less LS-supportive clinical data, albeit the LS-indicative tumor IHC finding. One sample consistently fell within the cut-off area interpreted as sample neither MMR proficient nor deficient, while another gave only an indicative result due to the shortage of tested material/assay repetitions. Interestingly, Patient 1 and one of their child (Patient 3), but not the other child (Patient 2) were found to carry a VUS (*MSH2* c.1805T>C (p.Leu602Pro). The DiagMMR results showing deficient MMR for Patient 1 and 3, but not for 2 facilitate the LS diagnosis while also suggesting, although not confirming, that the VUS is pathogenic. In agreement, recent results from a methylation tolerance-based assay (60) suggests that the p.Leu602Pro alteration is deleterious, and all information publicly available to date makes it justified to classify the variant as likely pathogenic (ACMG/ACP criteria PS3, PM1, PM2, PP2, and PP3 are met). These findings demonstrate the importance of the ability to test the other family members of LS individuals without tumor-derived information, but also demonstrate the DiagMMR's potential in assisting with VUS classification. Here, the DiagMMR results directly impacted the cancer surveillance plan of the seven patients participating in the clinical pilot.

The DiagMMR test is shown to be a novel, minimally invasive method for detecting LS causing deficient MMR function from constitutional tissue. With high specificity and sensitivity, it offers an excellent LS cancer prediction and prevention strategy. The test has currently been clinically validated for distinguishing normal MMR level from reduced MMR corresponding to pathogenic *MSH2* and *MSH6* variant carrier levels. In the near future, the quantitative nature of the DiagMMR method can even enable more detailed classification of the pathogenicity, as already depicted by patients’ variable distances to the assay cutoff (Fig. 4).
